# Prognostic Value of Exercise Testing in Patients with Liver Cirrhosis

**DOI:** 10.3390/diagnostics16071036

**Published:** 2026-03-30

**Authors:** Teresa John, Alexander Avian, Gabor Kovacs, Peter Fickert, Vasile Foris, Maximilian Gumpoldsberger, Nikolaus John, Antonia Laule, Horst Olschewski, Vanessa Stadlbauer, Nikolaus Kneidinger, Rudolf Stauber, Philipp Douschan

**Affiliations:** 1 Division of Respiratory Medicine, Department of Internal Medicine, Lung Research Cluster, ERN Pulmonary Hypertension National Expert Center, Medical University of Graz, 8036 Graz, Austria; 2 Institute for Medical Informatics, Statistics and Documentation, Medical University of Graz, 8010 Graz, Austria; 3 Division of Gastroenterology and Hepatology, Department of Internal Medicine, Medical University of Graz, 8010 Graz, Austria; 4 Channing Division of Network Medicine, Brigham and Women’s Hospital, Harvard Medical School, Boston, MA 02115, USA; 5 Faculty of Medicine, Sigmund Freud Private University, 1020 Vienna, Austria; 6 Department of Pneumology, Intensive Care Medicine and Sleep Medicine, Charité University Medicine, 10117 Berlin, Germany

**Keywords:** liver cirrhosis, exercise performance, prognosis, cardiopulmonary exercise testing, six-minute-walking distance

## Abstract

**Background/Objectives:** Cirrhosis is associated with increased mortality. In this study, we aimed to investigate the prognostic relevance of 6-min-walk-distance- and cardiopulmonary exercise testing (CPET)-derived peak oxygen uptake (VO_2_) as estimates of exercise capacity in outpatients with cirrhosis. **Methods:** Patients underwent a comprehensive clinical characterization including cardiopulmonary exercise testing, six-minute-walking-test-derived distance, and echocardiography. We stratified the cohort using established prognostic thresholds for the six-minute-walking-test-derived distance (440 m) and peak VO_2_ (65% predicted) and Child–Pugh class (A vs. B/C). Competing risk analyses were performed using cumulative incidence functions and subdistribution hazard models to assess the impact of baseline variables on mortality, accounting for liver transplantation (LT) as a competing event and for age and sex. The prognostic value of exercise performance was analyzed first, followed by the stepwise inclusion of additional variables; multicollinearity precluded a full multivariable model. **Results:** We enrolled 197 patients in Child–Pugh Class A, B, and C (*N* = 92, *N* = 80, *N* = 25 patients; male *N* = 146, age: 56 ± 9 years). During the observation time of 85 (25–105) months, 48 patients underwent a liver transplant, and 88 died. Both the six-minute-walking-test-derived distance ≤ 440 m (*p* = 0.002, sHR: 0.996 95% CI: 0.993–0.998) and peak VO_2_ ≤ 65% predicted (*p* = 0.023, sHR: 0.987 95% CI: 0.976–0.998) were strong independent predictors of mortality. While the six-minute-walking-test-derived distance consistently remained significant across most models, the peak VO_2_ retained significance only when adjusted for creatinine. Combining exercise capacity and the Child–Pugh classification identified patients at a particularly high mortality risk. **Conclusions:** In patients with liver cirrhosis outside the liver transplant setting, the impaired six-minute-walking-test-derived distance and peak VO_2_ serve as predictors of mortality and may help to identify patients at a particularly high mortality risk. These results suggest that functional capacity provides complementary information to established liver disease severity scores and could be considered in a multidimensional risk assessment approach in patients with liver cirrhosis.

## 1. Introduction

Patients with cirrhosis are often limited in their exercise performance [1]. Disease complicating conditions like cirrhotic cardiomyopathy, hepatopulmonary syndrome (HPS), or portopulmonary hypertension may significantly contribute to exercise limitation, causing impaired oxygen delivery and utilization during exertion [1,2,3]. Moreover, profound muscle wasting resulting in sarcopenia, which is highly prevalent in cirrhosis, further restricts daily activities and is an independent predictor of mortality and liver transplant outcomes [4,5,6,7]. The American Association for the Study of Liver Diseases highlights that frailty and sarcopenia should be routinely assessed using standardized tools such as the Liver Frailty Index, hand grip strength, and gait speed, as these are practical and prognostically relevant in ambulatory settings [7].

The cardiopulmonary-exercise-testing-derived peak oxygen uptake (peak VO_2_) and six-minute-walking-test-derived distance are established prognosticators in various conditions including chronic heart disease [8,9], chronic lung disease [10,11] and pulmonary hypertension [12,13]. A lower peak VO_2_ and shorter six-minute-walking distance are associated with increased mortality, a higher risk of decompensation, and poorer post- transplant outcomes [14]. While the six-minute-walking-test-derived distance is an easy, non-invasive, and widely available tool, cardiopulmonary exercise testing can provide more detailed insights about specific components of exercise limitation. Furthermore, physical activity interventions, including aerobic and resistance exercise, are safe and can improve muscle mass, strength, functional capacity, and quality of life in compensated cirrhosis [7,14,15]. The American Association for the Study of Liver Diseases recommends tailored exercise prescriptions and regular reassessment to optimize outcomes [7].

For patients on the liver transplant waiting list, exercise testing is considered as an established predictor of outcome [16,17]. However, few data exist regarding the underlying mechanisms of exercise limitation and the impact of physical capacity on long-term survival in outpatients with mild-to-moderate liver disease outside the liver transplant setting.

The aim of this study was to assess exercise capacity and its association with disease severity and long-term outcomes in patients with mild-to-moderate liver cirrhosis. In particular, we sought to evaluate the prognostic significance of the six-minute-walking-test-derived distance and peak VO_2_, and to determine whether combining functional parameters with established clinical scores, such as the Child–Pugh classification, helps to identify patients at a particular mortality risk.

## 2. Methods

### 2.1. Patients

We analyzed patients with liver cirrhosis from our prospective registry study undergoing evaluation for exercise limitations and pulmonary vascular disease. Consecutive patients from our liver outpatient clinic were prospectively enrolled between 2011 to 2016. All patients were screened for pulmonary vascular disease such as hepatopulmonary syndrome and portopulmonary hypertension and exercise limitations. Accordingly all study participants were invited to undergo cardiopulmonary exercise testing, six-minute-walking-test-derived distance, and resting and exercise echocardiography [1]. If clinically indicated, patients also underwent right heart catheter investigation. Liver cirrhosis was diagnosed based on liver histology or typical clinical, biochemical, and radiological findings. Exclusion criteria comprised comorbidities for exercise testing like the presence of pulmonary hypertension, except for portopulmonary hypertension, history of acute pulmonary embolism, myocardial infarction, esophageal variceal hemorrhage, severe infectious diseases within 6 months before study, hepatocellular carcinoma, liver transplantation, moderate-to-severe obstructive or restrictive pulmonary disease, left ventricular ejection fraction < 50%, refractory ascites, hepatorenal syndrome, persistent hepatic encephalopathy > grade I, and pregnancy. For this analysis, subjects with non-cirrhotic portal hypertension were also excluded. All patients provided written informed consent. Survival data were provided by Statistics Austria, the federal statistical institute of Austria. This study was approved by the local ethics committee at the Medical University of Graz (35-313 ex 22/23).

### 2.2. Clinical Evaluation and Exercise Testing

At baseline, all patients underwent clinical evaluation including cardiopulmonary exercise testing, six-minute-walking-test-derived distance, standard pulmonary function tests, blood gas analysis using ABL-800-FLEX blood gas analyzer (Fa. Drott, Wiener Neudorf, Austria), laboratory testing, resting echocardiography, and contrast enhanced echocardiography (Vivid E9 Ultrasound^®^ device by GE healthcare, Chicago, IL, USA) performed by trained staff using a conventional ultrasound system with a 2.5 MHz duplex transducer. Tricuspid regurgitation was used to estimate systolic pulmonary arterial pressure (sPAP) using simplified Bernoulli equation (sPAP = 4 × velocity^2^ + RAP), where RAP is the estimated right atrial pressure, as previously suggested [12,18]. Contrast enhanced echocardiography was performed by injection of agitated saline into the brachial vein to investigate the presence of hepatopulmonary syndrome, as previously described [1]. Hepatopulmonary syndrome was defined as the signs of intrapulmonary vascular dilatations and impaired gas exchange, as assessed by blood gas analysis (AaDO_2_ ≥ 15 mmHg or ≥20 mmHg for patients aged >64 years). Cardiopulmonary exercise testing was performed on a cycle ergometer (ER 900 EL, ergoline, Bitz, Germany) using a stepwise increasing workload with 25 Watt increase every 2 min. Complaints and other reasons for exercise cessation were noted after each cardiopulmonary exercise testing investigation. Patients pointed to a scale from 0–10 (0 = no symptom, 10 = most severe symptoms) to indicate dyspnea, general exhaustion, and other reasons for exercise termination. Peak VO_2_ was calculated as previously described [19]. A reduced exercise capacity was defined by a peak VO_2_ < 80% predicted. In case of a reduced peak VO_2_, cardiac, ventilatory, and metabolic limitation was defined by the amount of utilization of the heart rate, ventilatory, and base excess reserves, respectively.

### 2.3. Statistics

Data are expressed as means ± standard deviation (SD) or median and interquartile range (IQR) for continuous variables. Categorical data are presented as absolute and relative frequencies. For in-between group comparisons, ANOVA and Mann–Whitney U-Tests were used, as appropriate. Categorical variables were analyzed using Fisher’s exact test or Chi2 test. Pearson and Spearman correlations were performed for parametric and non-parametric variables, respectively. We used established predictive cutoff values of six-minute-walking-test-derived distance (440 m) and peak VO_2_ (65% predicted) used in the field of cardiopulmonary and pulmonary vascular disease [12] to split the group into two and further subdivided them by Child–Pugh Score A vs. Child–Pugh Score B + C.

Cumulative incidence functions (CIFs) were used to show the proportions of patients with the event of interest (death) or the competing event (liver transplantation, LT). To analyze the effect of baseline characteristics on CIFs, the proportional subdistribution hazard regression described by Fine and Gray [20] was used and subdistribution hazard ratios (sHRs) were calculated. In a first step, the impact of six-minute-walking-test-derived distance and peak VO_2_ was analyzed. In a second step, additional baseline variables (Child–Pugh Stage, MELD score, bilirubin, albumin, International Normalized Ratio (INR), creatinine, and N-terminal pro-brain natriuretic peptide (NT-proBNP)) were added individually to the two models. Due to multicollinearity reasons, no overall model was calculated. Multicollinearity was assessed using variance inflation factor (VIF). In each analysis, sex and age were included as covariates. Prediction models including MELD or Child–Pugh class, adjusted for age and sex, were compared with age- and sex-adjusted models including 6MWD or peak VO_2_. Time-adjusted ROC curves were performed to check for the additive predictive value of 6MWD and peak VO_2_ compared to MELD and Child–Pugh Class. A *p*-value < 0.05 was considered significant. Statistical analysis was performed using SAS 9.4 (Copyright (c) 2016 by SAS Institute Inc., Cary, NC, USA).

## 3. Results

### 3.1. Patients

A total of 197 patients met all the inclusion and exclusion criteria and were analyzed. At the time of enrolment, the patients were 56 ± 10 years old and the majority were male. Most patients were in Child–Pugh Stage A and Child–Pugh Stage B (92 (47%) and 80 (41%)), while only 25 were in Child–Pugh Stage C (13%)). Dyspnea on exertion was quite common but mostly mild. Overall, the patients had normal lung function and cardiac performance. Laboratory testing and further patient characteristics are provided in Table 1. The underlying etiology of cirrhosis was alcohol (60%), Hepatitis C Virus Infection (16%), Metabolic-Dysfunction-Associated Steatotic Liver Disease (MASLD) (6%), and autoimmune hepatitis (6%) (Supplemental Table S1). Furthermore, *N* = 54 (27%) patients were diagnosed with hepatopulmonary syndrome and five (2.5%) with portopulmonary hypertension. Supplemental Table S2 shows the comorbidities.

### 3.2. Exercise Performance

**Figure 1:**
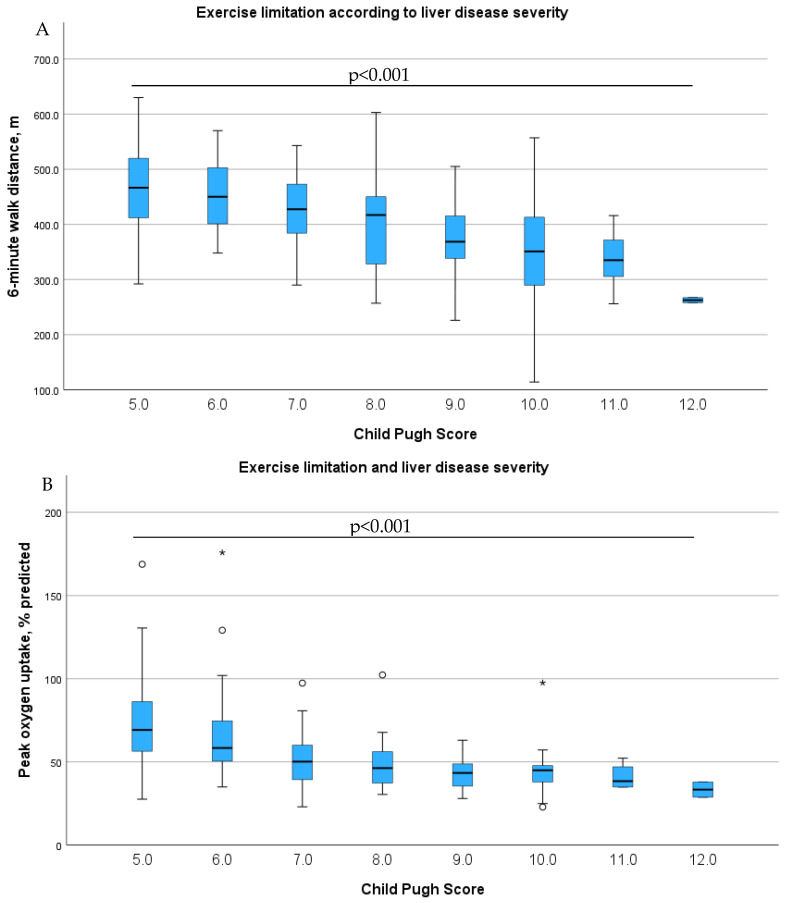
Boxplots showing that 6-min-walk distance (**A**) and peak oxygen uptake (**B**) are continuously decreasing with liver disease severity. ° Moderate outliers, meaning values that lie somewhat outside the typical range (by definition, between 1.5 × IQR and 3 × IQR from the quartile); * Extreme outliers, meaning values that lie far outside the typical range (by definition, more than 3 × IQR from the quartile).

All patients underwent at least one type of exercise testing. The six-minute-walking-test-derived distance was performed in *N* = 177 patients and cardiopulmonary exercise testing in *N* = 170 patients, and *N* = 150 underwent both tests. Overall, the patients were markedly reduced in their daily exercise performance with 422 ± 79 m in the six-minute-walking-test-derived distance and a peak VO_2_ of 54 (42–70) % predicted (Table 1), and the impairment in exercise capacity was significantly associated with liver disease severity (peak VO_2_% predicted: Child–Pugh A 71% (57–92), Child–Pugh B 50% (40–60), Child–Pugh C 42% (35–54), *p* < 0.001; and six-minute-walking-test-derived distance: Child–Pugh A 459 ± 73 m, Child–Pugh B 402 ± 81 m, Child–Pugh C 342 ± 93 m, *p* < 0.001) (Figure 1).

Subjective and objective exercise-limiting factors:

At cessation of exercise, subjects reported muscular exhaustion or muscle pain (65%), dyspnea (13%), or other reasons (9%) as the main exercise-limiting factor. Chest pain was rare (3%). As a major cause of exercise limitation, we identified ventilatory (41%) and metabolic (34%) factors. Cardiac limitation was rare (6%) (Table 2).

### 3.3. Exercise Performance as Predictor of Survival

During the median (IQR) observation time of 85 (25–105) months, a total of 48 (24%) patients underwent liver transplantation and 88 patients (59%) died. Mortality rates were significantly associated with increasing liver disease severity: Child–Pugh A 45% (38 of 85), Child–Pugh B 75% (38 of 51), and Child–Pugh C 87% (13 of 15). The leading cause of death was liver failure (*n* = 46, 52%), hepatocellular carcinoma (*N* = 10, 11%), sepsis (*N* = 9, 10%), and esophageal varices bleeding (*N* = 6; 7%). In *n* = 8 (9%), the cause of death remained unknown. Stratifying the cohort by the predefined cutoff values, we found that patients with a six-minute-walking-test-derived distance ≤ 440 m and a peak VO_2_ ≤ 65% predicted had significantly worse liver function, more dyspnea, a higher body mass index (BMI), a lower vital capacity, and higher cardiac index values as compared to those with a preserved six-minute-walking-test-derived distance and peak VO_2_ (Table 1). Out of the 88 patients who died, *n* = 70 (80%) had a low six-minute-walking-test-derived distance ≤ 440 m and *n* = 60 (68%) had a peak VO_2_ ≤ 65% predicted.

The cumulative incidence of death, while accounting for liver transplantation, was higher with increasing age (*p* = 0.008; sHR 1.03 95% CI: 1.01–1.06), but was not affected by sex (*p* = 0.919; sHR 1.03 95% CI: 0.63–1.62). Exercise markers had an age- and sex-independent impact on survival (six-minute-walking-test-derived distance; *p* = 0.002, sHR: 0.996 95% CI: 0.993–0.998 and peak VO_2_ *p* = 0.023, sHR: 0.987 95% CI: 0.976–0.998).

In outcome models including age, sex, and one of the baseline parameters such as albumin, creatinine, NT-proBNP, bilirubin, INR, MELD, and Child–Pugh score, all models with the six-minute-walking-test-derived distance were significant except for models including the MELD score and Child–Pugh score. In contrast, peak VO_2_ only remained a significant predictor when adjusted for age, sex, and creatinine (Table 3).

**Figure 2:**
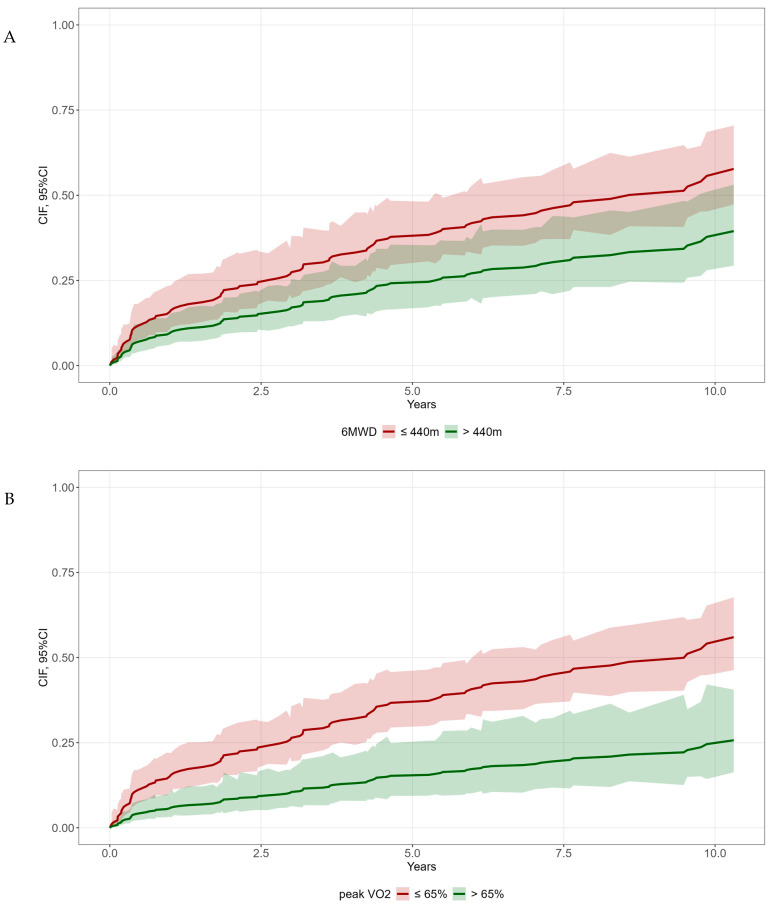
Cumulative incidence function overall survival and liver transplantation (competing risk) adjusted for age and sex by (**A**) six-minute-walking distance (6MWD) (*p* = 0.020); (**B**) peak VO_2_ of ≤ vs. >65% predicted (*p* = 0.001). Abbreviations: 6MWD: 6-min-walk distance. Peak VO_2_: peak oxygen uptake.

After dichotomization and adjustment for age and sex, both the six-minute-walking-test-derived distance (*p* = 0.020, sHR: 0.58, 95% CI: 0.37–0.92; Figure 2A) and peak VO_2_ (*p* = 0.001, sHR: 0.36, 95% CI: 0.20–0.64; Figure 2B) remained significant predictors of mortality.

**Figure 3:**
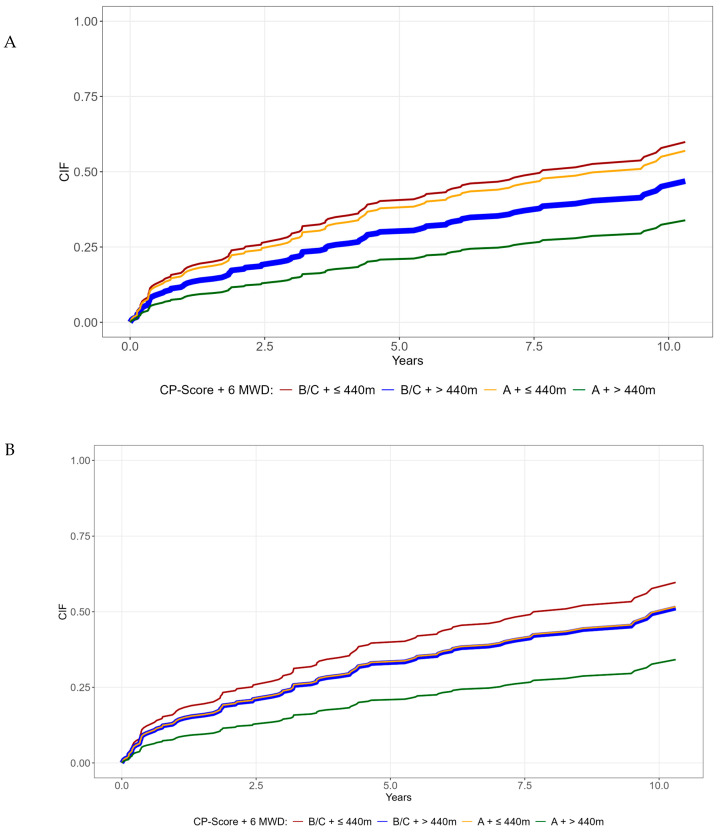
Cumulative incidence function overall survival and liver transplantation (competing risk) for (**A**) combined CP score/6MWD groups (Group 1: Child–Pugh B,C 6MWD ≤ 440 m (red line); group 2: Child–Pugh B,C, 6MWD > 440 m (orange line); group 3: Child–Pugh A, 6 MWD ≤ 440 m (blue line); Reference: Child–Pugh A, 6 MWD > 440 m (green line); group 1 vs Reference: *p* = 0.006, group 2 vs. Reference, *p* = 0.307., group 3 vs. Reference: *p* = 0.016). (**B**) In the multivariate model, correcting for age and sex (group 1 vs Reference: *p* = 0.008, group 2 vs. Reference, *p* = 0.196, group 3 vs. Reference: *p* = 0.065).

Combining the dichotomized six-minute-walking-test-derived distance with the Child–Pugh class identified subgroups with an additional mortality risk (Figure 3A). Patients with Child–Pugh Class A and a six-minute-walking-test-derived distance ≤ 440 m had worse outcomes than those with Class A and a six-minute-walking-test-derived distance > 440 m (*p* = 0.016, sHR: 2.04, 95% CI: 1.14–3.64), although this difference was not significant after adjustment for age and sex (*p* = 0.065) (Figure 3B). Interestingly, these patients had similar survival to those with Child–Pugh Class B + C and a six-minute-walking-test-derived distance > 440 m (Figure 3B).

Comparing prediction models including MELD or CP (adjusted for age and sex) showed no significant different predictive value compared to models including 6MWD (Supplementary Table S3A,B) or peak VO_2_ (Supplementary Table S4A,B). Moreover, the time-dependent AUC did not differ (Supplementary Table S3A,B and Supplementary Table S4A,B).

## 4. Discussion

This study aimed to evaluate the clinical impact and prognostic value of exercise capacity, assessed by six-minute-walking-test-derived distance and peak VO_2_, in patients with mild-to-moderate liver cirrhosis. Our findings suggest that both the reduced six-minute-walking-test-derived distance and peak VO_2_ are strong predictors of mortality, even after adjustment for age and sex. The combination of the six-minute-walking-test-derived distance and Child–Pugh classes identified patients at a particularly high mortality risk.

There is broad evidence that patients with liver cirrhosis are limited in their exercise capacity and that this limitation is associated with liver disease severity [1,15]. Wiesinger et al. performed a study in 26 patients with cirrhosis and found that they were impaired in their daily activities, which was also associated with cirrhosis severity [21]. Terziyski et al. found in 19 patients with mild-or-moderate liver cirrhosis a reduced exercise tolerance dependent on liver disease severity [22]. Our prospective large study, including 197 outpatients, confirmed these findings. The pathophysiology of impaired exercise capacity in patients with cirrhosis may be multifactorial and is still not fully understood. Up to one-third of patients with cirrhosis develop cirrhotic cardiomyopathy, characterized by impaired left ventricular contractility, diastolic dysfunction, and electrophysiological alterations [23,24]. Interestingly, at rest, patients with cirrhotic cardiomyopathy typically show a hyperdynamic circulation with decreased systemic arterial pressures due to vasomotor collapse. During exercise, however, the cardiac output response is nearly completely blunted, which, per se, limits exercise capacity [25,26]. In addition, pulmonary vascular complications of liver cirrhosis, including hepatopulmonary syndrome and portopulmonary hypertension, are associated with an impaired exercise capacity [27,28]. Hepatopulmonary syndrome is characterized by a loss of pulmonary vascular tone and, accordingly, ventilation perfusion mismatch, causing systemic hypoxemia [28,29]. The prevalence has been estimated at up to 33% in patients with cirrhosis [1]. Portopulmonary hypertension, in transplant candidates, has a prevalence of 4–16% and is caused by the constrictive remodeling of pulmonary arteries leading to an increased pulmonary vascular resistance and right ventricular strain [1,12]. In our collective, *N* = 40 patients had an increased cardiac output as an indicator for a hyperdynamic state. None of these patients showed a clinically relevant left ventricular dysfunction. However, this may be due to the fact that relevant left ventricular dysfunction was an exclusion criterium for our study. In our few patients with portopulmonary hypertension, exercise limitation by a circulatory limitation was caused in 3 patients and a metabolic limitation in 2 of the overall cohort. The presence of ascites may further limit exercise capacity by ventilatory limitation due to restrictive ventilation caused by the abdominal compression of the lung [30]. Indeed, 36 patients had this restrictive ventilation pattern and 35 of them had ascites.

In addition to cardiopulmonary exercise limitation, up to 68% of cirrhotic patients suffer from frailty, which is defined as a syndrome of decreased reserve and resistance to stressors that results in multiple organ dysfunction including skeletal muscle and causing vulnerability to adverse outcomes [6,7]. Frailty is strongly associated with impaired exercise tolerance [5].

This study is the first to analyze exercise-limiting factors in patients with cirrhosis. Interestingly, among the subjective factors, muscle exhaustion and muscle pain were the most frequent reasons for exercise termination, while dyspnea and cardiac complaints were rare (3%). Among the objective exercise-limiting factors, ventilatory (41%) and metabolic factors (34%) were predominant and cardiac factors remained rare. This result may be biased by the fact that patients with severe cardiac comorbidities were excluded. Therefore, this population might underestimate the true prevalence of cardiac exercise limitation in an unselected cohort. A total of 29 patients exhibited combined exercise-limiting factors, and, notably, all of them reported muscular weakness/pain as their primary reason for exercise cessation. This finding suggests that frailty and, particularly, peripheral muscle dysfunction may represent a major limiting factor in this subset of patients. However, neither frailty nor functional performance measures are currently integrated into liver disease severity scores, despite their importance for transplant-free survival and post-transplant outcomes [31,32,33]. This may be due to the fact that frail patients and those with a reduced functional status may lack the physiological reserves to survive the transplant procedure. We acknowledge that frailty and sarcopenia are important determinants of both exercise capacity and prognosis in patients with cirrhosis. The lack of formal frailty assessments in our cohort represents a potential confounder, as it may partly account for the observed associations between 6MWD, peak VO_2_, and clinical outcomes. Future studies incorporating standardized assessments of frailty and sarcopenia are needed to better delineate their independent prognostic contributions. Taken together, multiple factors including frailty- and cirrhosis-associated comorbidities such as ventilatory limitation due to increased intra-abdominal pressure contribute to an impaired exercise capacity in patients with cirrhosis.

Data from the literature indicate that a reduced exercise tolerance is associated with a worse outcome in the liver transplant setting [34]. Epstein et al. performed a prospective study in 156 patients with cirrhosis on the liver transplant waiting list and found that a reduced peak VO_2_ (<60% predicted) was associated with short-term survival after liver transplantation [34]. In another prospective study by Dharancy et al., patients with cirrhosis waiting for liver transplantation had a significantly lower 1-year survival (65%) when the peak VO_2_ was <60% predicted compared to those with a peak VO_2_ exceeding 60% predicted (96%) [35]. Ow et al. investigated the association between functional capacity and short-term survival before liver transplantation in 176 patients. They found the peak VO_2_ to be a predictor of short-term survival, adjusted for the MELD score, UK Model for End-Stage Liver Disease (UKELD) score, age, and anaerobic threshold [36]. In a study of 106 outpatients with all stages of liver cirrhosis without relevant cardiopulmonary comorbidities, the six-minute-walking-test-derived distance was an independent predictor of mortality when adjusted for the Child–Pugh score [37]. A distance of 387 m (sensitivity 91%, specificity 88%) was associated with mortality.

Few data exist on the prognostic relevance of exercise performance in compensated cirrhosis not listed for liver transplantation. In a prospective study of 55 patients with compensated cirrhosis, a six-minute-walking-test-derived distance of 402 m turned out to be a significant predictor of clinical decompensation. The authors suggested to add the six-minute-walking-test-derived distance to the clinical assessment of these patients [38]. However, survival was not investigated.

Established prognostic tools such as the MELD and Child–Pugh scores are widely used to predict disease progression and transplant need [30]. However, these scores do not account for physical performance, an important determinant of the ability to tolerate peri-transplant stress and complications [34,35,36]. Our findings extend previous observations by demonstrating that a reduced exercise capacity, assessed by both the six-minute-walking-test-derived distance and peak VO_2_, are age- and sex-independent predictors of long-term mortality in patients with mild-to-moderate liver cirrhosis. In our cohort, a six-minute-walking-test-derived distance ≤ 440 m or a peak VO_2_ ≤ 65% predicted were significantly associated with poor liver function, dyspnea, and poor survival compared to those with better functional performance. Importantly, both the six-minute-walking-test-derived distance and peak VO_2_ remained significant prognostic markers even after adjusting for age and sex, while other baseline variables such as albumin, creatinine, NT-proBNP, and INR did not add prognostic value and were even outperformed when combined with markers of exercise performance. Interestingly, adding the six-minute-walking-test-derived distance to established risk scores (Child–Pugh score and MELD score) did not result in prediction improvement. This finding suggests that exercise capacity explains the outcome heterogeneity in a manner partly overlapping with the clinical severity captured by these scores. In our models, approximately 16% of the variance was shared between the six-minute-walking-test-derived distance and the cumulative scores, indicating a meaningful but not complete overlap without violating multicollinearity assumptions. Notably, in several adjusted models, the 6MWD remained a more robust predictor than peak VO_2_, which lost statistical significance in multiple analyses. Of note, a direct comparison of the Child–Pugh class or MELD with peak VO_2_ and 6MWD showed no differences in the prognostic value of exercise tests as compared to these established biochemical risk prediction scores. These data suggest that the simpler and more widely accessible 6MWD may represent a pragmatic and clinically sufficient tool for risk stratification in clinical routine. Moreover, the combination of the dichotomized six-minute-walking-test-derived distance with established risk scores identified particularly high-risk subgroups within CP stages. This supports the prior findings by Ow et al. [36], who showed that the peak VO_2_ independently predicts short-term survival even after adjusting for the MELD and UKELD scores. However, after adjustment for age and sex, these differences did not remain significant (*p* = 0.065). Both the reduced six-minute-walking-test-derived distance and peak VO_2_ were age- and sex-independent predictors of mortality, and their combination further improved risk stratification, identifying patients with a more-than-threefold-higher risk of death. Notably, a preserved peak VO_2_ appeared slightly more protective than the six-minute-walking-test-derived distance alone when the two measures were discordant.

Taken together, these results suggest that functional capacity, as assessed by simple tests such as the six-minute-walking-test-derived distance or cardiopulmonary exercise testing, provides complementary information to established severity scores and could be considered in a multidimensional risk assessment approach in patients with liver cirrhosis.

## 5. Limitations

The single-center character is a limitation of the present study. However, the cohort size and the number of events was high enough to allow for a multivariate survival analysis correcting for covariates like age, gender, Child–Pugh score, and MELD score, as well as bilirubin, creatine, International Normalized Ratio (INR), albumin, and NT-proBNP. Due to multicollinearity reasons, no overall model was calculated. Another limitation might be the fact that not all of the patients underwent both cardiopulmonary exercise testing and the six-minute-walking test; however, this was only the case in 10% of patients. Furthermore, 17% of the patients had not reached any objective exercise-limiting factor at cessation of exercise testing, suggesting motivational or peripheral factors. Another limitation is the lack of sarcopenia and frailty markers as part of our dataset.

## 6. Conclusions

In well-characterized outpatients with mostly compensated cirrhosis, cardiopulmonary exercise capacity is severely limited and significantly associated with poor survival. The reduced six-minute-walking-test-derived-distance and impaired peak VO_2_ independently predict mortality. The combination of functional parameters may further refine risk stratification and help to identify subgroups at particular risk. A functional assessment, when integrated with liver disease severity scores, provides additional prognostic information and may guide clinical decision-making.

## Figures and Tables

**Table 1:** Patient characteristics.

Variables	Total	Peak VO_2_ > 65%	Peak VO_2_ < 65% Pred.	*p*-Value *	6MWD > 440 m	6 MWD < 440 m	*p*-Value t
	*N* = 197	Pred. *N* = 53	*N* = 144		*n* = 75	*n* = 101	
Male/Female	146/51	29/24	117/27	*p* < 0.001	65/19	73/28	*p* = 0.050
Age, years	56 ± 9	58 ± 10	55 ± 9	*p* = 0.097	54 ± 11	56 ± 9	*p* = 0.061
Body mass index, kg/m^2^	27 ± 5	26 ± 3	28 ± 5	*p* = 0.006	26 ± 4	28 ± 5	*p* = 0.035
Child Pugh Score	6 (5–8)	5 (5–6)	8 (6–9)	*p* < 0.001	5 (5–7)	8 (6–9)	*p* < 0.001
MELD Score	12 (9–17)	9 (7–11)	15 (10–18)	*p* < 0.001	13 (9–18)	18 (15–23)	*p* < 0.001
Child Pugh Stage *N* = 197							
A	92 (47%)	46 (87%)	46 (32%)	*p* < 0.001	53 (71%)	32 (32%)	*p* < 0.001
B	80 (40%)	5 (9%)	74 (51%)		20 (27%)	49 (48%)	
C	25 (13%)	1 (4%)	24 (17%)		2 (2%)	20 (20%)	
WHO-Functional class *N* = 196							
I	93 (48%)	33 (62%)	60 (42%)	*p* < 0.001	53 (71%)	32 (32%)	*p* < 0.001
II	95 (48%)	19 (36%)	76 (53%)		22 (29%)	61 (61%)	
III	8 (4%)	1 (2%)	7 (5%)			7 (7%)	
Laboratory Testing							
Hemoglobin, g/dL	11.8 ± 1.9	13.9 ± 2.0	11.8 ± 2.2	*p* < 0.001	13.7 ± 1.9	11.6 ± 2.3	*p* < 0.001
Creatinine, mg/dL	0.8 ± 0.2	0.89 ± 0.26	0.96 ± 0.35	*p* = 0.204	0.9 ± 0.2	1.0 ± 0.4	*p* = 0.008
Bilirubin, mg/dL	2.5 ± 2.0	1.3 ± 1.4	4.2 ± 6.0	*p* < 0.001	2.5 ± 4.7	3.8 ± 4.3	*p* = 0.057
NT-proBNP, pg/mL	181 ± 125	86 ± 72	209 ± 361	*p* < 0.001	92 ± 85	250 ± 418	*p* = 0.002
INR	1.4 ± 0.4	1.2 ± 0.2	1.5 ± 0.3	*p* < 0.001	1.3 ± 0.3	1.5 ± 0.3	*p* < 0.001
Albumin, g/dL	3.3 ± 1.1	4.2 ± 0.5	3.6 ± 0.7	*p* < 0.001	4.0 ± 0.7	3.6 ± 0.7	*p* < 0.001
GFR, mL/min	93 ± 12	86 ± 21	87 ± 25	*p* = 0.928	94 ± 19	82 ± 25	*p* = 0.001
Lung function testing							
FVC, % predicted	84 ± 11	107 ± 14	90 ± 15	*p* < 0.001	100 ± 14	91 ± 17	*p* < 0.001
FEV1, % predicted	81 ± 13	100 ± 15	85 ± 16	*p* < 0.001	95 ± 14	86 ± 18	*p* < 0.001
FEV1/FVC	76 ± 5	76 ± 6	76 ± 7	*p* = 0.484	76 ± 6	77 ± 7	*p* = 0.520
TLC, % predicted	93 ± 13	104 ± 13	95 ± 13	*p* < 0.001	100 ± 13	94 ± 14	*p* = 0.024
DLCOcSB, % predicted	72 ± 16	90 ± 16	74 ± 15	*p* < 0.001	86 ± 18	75 ± 15	*p* < 0.001
DLCOcVA, % predicted	87 ± 21	91 ± 17	85 ± 18	*p* = 0.029	91 ± 18	86 ± 16	*p* = 0.051
Echocardiographic parameters							
Cardiac ouput, mL/min	6.6 ± 2.2	5.6 ± 1.4	6.5 ± 1.9	*p* = 0.010	5.9 ± 1.5	6.4 ± 2.0	*p* = 0.141
TAPSE, mm	28 ± 5	25 ± 4	26 ± 5	*p* = 0.028	25 ± 5	26 ± 5	*p* = 0.096
sPAP, mmHg	33 ± 5	27 ± 5	30 ± 9	*p* = 0.023	27 ± 7	31 ± 9	*p* = 0.012
TAPSE/sPAP, mm/mmHg	0.85 ± 0.13	0.93 ± 0.28	0.95 ± 0.29	*p* = 0.745	0.95 ± 0.26	0.92 ± 0.27	*p* = 0.351
Exercise testing							
6MWD, m	422 ± 79	478 ± 77	399 ± 82	*p* < 0.001	502 ± 46	363 ± 61	*p* < 0.001
Peak Exercise level, Watt	75 (50–100)	100 (75–150)	75 (50–100)	*p* < 0.001	100 (75–150)	75 (50–100)	*p* < 0.001
Peak VO_2_, % predicted	54 (42–70)	97 (85–119)	55 (46–60)	*p* < 0.001	67(54–89)	46 (37–56)	*p* < 0.001
VO_2,max_, mL/min·kg	17 (13–21)	23 (20–26)	15 (13–18)	*p* < 0.001	21 (17–25)	14 (13–17)	*p* < 0.001

Definition of abbreviations: NT-proBNP = N-terminal pro-brain natriuretic peptide, INR = international normalized ratio, GFR = glomerular filtration rate, FVC = forced vital capacity, FEV1 = forced expiratory volume in the first record of expiration, TLC = total lung capacity, DLCOc = diffusing capacity of lung for carbon monoxide; SB = single-breath, DLCOcVA = diffusing capacity of lung for carbon monoxide for alveolar volume corrected for hemoglobin, TAPSE = tricuspid annular plane systolic excursion, sPAP = systolic pulmonary arterial pressure, 6MWD = 6-min-walk distance, VO_2_ = oxygen uptake in percent predicted, VO_2,max_ = absolute maximum oxygen uptake. Normally distributed values are expressed as mean ± SD; non-parametric variables are expressed as median and interquartile range; * comparison between the two groups of peak VO_2_ > 65% predicted and ≥65% predicted, respectively; ^t^ comparison between the two groups of 6MWD ≥ 440 m and <440 m, respectively.

**Table 2:** Reasons for exercise limitation.

Reasons for Exercise Termination (Subjective)	Total *N* = 170	Exercise Limitation (Objective)	Total *N* = 170
Muscle weakness	128 (75%)	Metabolically	57 (34%)
Pulmonary	25 (15%)	Ventilatory	70 (41%)
Cardiac	5 (3%)	Cardiac	11 (6%)
Others	12 (7%)	Others	32 (19%)

Subjective: pulmonary reasons include dyspnea and cough; cardiac reasons include blood pressure (too low, or too high), angina pectoris, and cardiac arrhythmia; other reasons include thirst, problems with the mouthpiece, and pain due to the position on the ergometer. Objective: other reasons include reached individual peak exercise in percent predicted: *N* = 13, and premature termination of exercise without objective reasons: *N* = 29.

**Table 3:** Competing risk analysis for prediction of death (competing risk: liver transplantation) by 6MWD (left) and peak VO_2_ (right), respectively.

6MWD	Significance	Subdistribution Hazard Ratios	Peak VO_2_	Significance	Subdistribution Hazard Ratios
Model 1a			Model 1b		
Sex	0.650	1.21 (0.67–1.81)	Sex	0.266	1.38 (0.77–2.38)
Age	0.017	1.03 (1.01–1.06)	Age	0.004	1.04 (1.01–1.07)
**6MWD**	**0.020**	**0.997 (0.994–0.999)**	Peak VO_2_	0.141	0.99 (0.98–1.00)
ln (Bilirubin)	0.079	1.24 (0.97–1.56)	ln (Bilirubin)	0.016	1.33 (1.05–1.67)
Model 2a			Model 2b		
Age	0.627	1.13 (0.67–1.85)	Sex	0.229	1.42 (0.79–2.47)
Sex	0.036	1.03 (1.00–1.06)	Age	0.008	1.04 (1.01–1.07)
**6MWD**	**0.011**	**0.996 (0.994–0.999)**	Peak VO_2_	0.064	0.99 (0.98–1.00)
Albumin	0.250	0.81 (0.55–1–16)	Albumin	0.735	0.93 (0.62–1.39)
Model 3a			Model 3b		
Sex	0.446	1.22 (0.72–1.98)	Sex	0.148	1.52 (0.85–2.63)
Age	0.028	1.03 (1.00–1.06)	Age	0.008	1.04 1.01–1.07)
**6MWD**	**0.015**	**0.997 (0.994–0.999)**	Peak VO_2_	0.139	0.99 (0.98–1.00)
INR	0.064	1.81 (0.93–3.27)	INR	0.026	2.02 (1.05–3.64)
Model 4a			Model 4b		
Sex	0.987	1.00 (0.58–1.66)	Sex	0.322	1.35 (0.73–2.40)
Age	0.046	1.03 (1.00–1.05)	Age	0.006	1.04 (1.01–1.07)
**6MWD**	**0.001**	**0.996 (0.993–0.998)**	**Peak VO_2_**	**0.021**	**0.99 (0.98–1.00)**
Creatinine	0.498	0.78 (0.36–1.52)	Creatinine	0.555	0.79 (0.35–1.62)
Model 5a			Model 5b		
Sex	0.790	0.93 (0.52–1.57)	Sex	0.459	1.27 (0.66–2.32)
Age	0.049	1.03 (1.00–1.06)	Age	0.030	1.03 (1.00–1.07)
**6MWD**	**0.007**	**0.996 (0.993–0.999)**	Peak VO_2_	0.077	0.99 (0.98–1.00)
ln (T-tproBNP)	0.839	1.03 (0.78–1.38)	ln (NT-proBNP)	0.363	1.14 (0.86–1.53)
Model 6a			Model 6b		
Sex	0.275	1.35 (0.78–2.26)	Sex	0.083	1.66 (0.92–2.89)
Age	0.027	1.03 (1.00–1.06)	Age	0.010	1.04 (1.01–1.07)
6MWD	0.054	0.997 (0.994–1.000)	Peak VO_2_	0.212	0.993 (0.981–1.004)
MELD	0.098	1.08 (0.99–1.19)	MELD	0.040	1.10 (1.01–1.21)
Model 7a			Model 7b		
Sex	0.607	1.14 (0.68–1.84)	Sex	0.249	1.40 (0.78–2.42)
Age	0.020	1.03 (1.01–1.06)	Age	0.007	1.04 (1.01–1.07)
6MWD	0.067	0.997 (0.994–1.000)	Peak VO_2_	0.207	0.99 (0.98–1.00)
CP class	0.064	1.14 (0.99–1.30)	CP class	0.058	1.14 (1.00–1.31)

Abbreviations: 6MWD: 6-minute-walk distance, VO_2_: oxygen uptake, CP: Child–Pugh.

## Data Availability

The data presented in this study are available on request from the corresponding author. The data are not publicly available due to legal reasons.

## Supplementary Materials

The following supporting information can be downloaded at: https://www.mdpi.com/article/10.3390/diagnostics16071036/s1, Table S1: etiology of cirrhosis; Table S2: comorbidities; Table S3A: Model comparison Child Pugh Class vs. 6MWT; Table S3B: Model comparison MELD vs. 6MWT; Table S4A: Model comparison Child Pugh Class vs. peak VO_2_; Table S4B: Model comparison MELD vs. peak VO_2_.

## Abbreviations

The following abbreviations are used in this manuscript:

AaDO_2_	Alveolar–arterial oxygen difference
AIH	Autoimmune Hepatitis
ANOVA	Analysis of Variance
BMI	Body Mass Index
CI	Confidence Interval
CIF	Cumulative Incidence Function
CP	Child–Pugh
CPET	Cardiopulmonary Exercise Testing
FVC	Forced Vital Capacity
HCV	Hepatitis C Virus
HPS	Hepatopulmonary Syndrome
IQR	Interquartile Range
INR	International Normalized Ratio
LT	Liver Transplantation
MASLD	Metabolic-dysfunction-associated steatotic liver disease
MELD	Model for End-Stage Liver Disease
mmHg	Millimeters of Mercury
N/A	Not Applicable
NT-proBNP	N-terminal pro-B-type Natriuretic Peptide
RAP	Right Atrial Pressure
sHR	Subdistribution Hazard Ratio
sPAP	Systolic Pulmonary Arterial Pressure
SD	Standard Deviation
VO_2_	Oxygen Uptake
WHO-FC	World Health Organization—Functional Class
6MWD	Six-Minute-Walk Distance
