# Supplementary material for: Prognostic Value of Exercise Testing in Patients with Liver Cirrhosis
Source: Diagnostics (Basel). 2026 Mar 30;16(7):1036. doi: 10.3390/diagnostics16071036 (PMC13072901; doi:10.3390/diagnostics16071036)
Supplement: Supplementary file 1 [file diagnostics-16-01036-s001.zip › diagnostics-4168916-supplementary.pdf]

Supplements

Supplement Table S1

Etiology of liver cirrhosis (partly overlapping)	Number (%)
ALD	118 (60%)
HCV	31 (16%)
MASLD	12 (6%)
AIH	11 (6%)
others	28 (14%)

Table S 1 Abbreviations: AIH = autoimmune hepatitis. ALD = alcoholic liver disease. MASLD = Metabolic Dysfunction-Associated Steatosis Liver Disease. HCV = hepatitis-C-virus; others consist of hepatitis-B Virus (N=4). Morbus Wilson (N=2). primary biliary cholangitis (N=1). primary sclerosing cholangitis (N=2). Both alcoholic and non-alcoholic steatohepatitis (N=2). secondary sclerosing cholangitis (N=1). biliary atresia (N=1). toxic agents (N=1). hemochromatosis (N=4). cryptogenic (N=8). biliary cholestasis (N=1). alpha-1 antitrypsin deficiency (N=1).

Supplement Table S2

Table S 2. Complications of liver cirrhosis at baseline.

Comorbidities associated with cirrhosis	Number (%)
Intrapulmonary Vascular Dilatations	70 (36%)
Hepatopulmonary syndrome	54 (27%)
Portopulmonary hypertension	5 (3%)
Hepatorenal syndrome	90 (46%)
Cirrhotic cardiomyopathy	53 (27%)

Supplement Table S3A

Model 1: Child Pugh Class; Model 2: 6MWD

model	*time*	*AUC*	*lower*	*upper*	*Delta_AUC*	*Lower_CI*	*Upper_CI*	*p_value*
Model 1	2.5	0.725	0.630	0.821				
Model 2	2.5	0.697	0.605	0.789	-0.028	-0.105	0.049	0.471
Model 1	5	0.657	0.570	0.744				
Model 2	5	0.651	0.567	0.735	-0.007	-0.077	0.063	0.853
Model 1	7.5	0.659	0.575	0.744				
Model 2	7.5	0.681	0.598	0.763	0.021	-0.051	0.093	0.562
Model 1	10	0.646	0.537	0.755				
Model 2	10	0.670	0.557	0.782	0.023	-0.064	0.111	0.602

Supplement Table S3B

Model 1: MELD ; Model 2: 6MWD

model	*time*	*AUC*	*lower*	*upper*	*Delta_AUC*	*Lower_CI*	*Upper_CI*	*p_value*
Model 1	2.5	0.709	0.614	0.804				
Model 2	2.5	0.698	0.605	0.790	-0.011	-0.104	0.082	0.820
Model 1	5	0.639	0.551	0.726				
Model 2	5	0.639	0.554	0.724	0.000	-0.078	0.078	0.998

Model 1	7.5	0.662	0.577	0.746				
Model 2	7.5	0.678	0.595	0.761	0.016	-0.059	0.091	0.674
Model 1	10	0.646	0.535	0.756				
Model 2	10	0.665	0.552	0.778	0.020	-0.072	0.111	0.675

Supplement Table S4A

Model 1: Child Pugh Class ; Model 2: peak VO_2

model	*time*	*AUC*	*lower*	*upper*	*Delta_AUC*	*Lower_CI*	*Upper_CI*	*p_value*
Model 1	2.5	0.713	0.617	0.809				
Model 2	2.5	0.653	0.555	0.752	-0.060	-0.136	0.017	0.125
Model 1	5	0.651	0.563	0.739				
Model 2	5	0.631	0.542	0.720	-0.020	-0.087	0.048	0.565
Model 1	7.5	0.640	0.554	0.726				
Model 2	7.5	0.669	0.584	0.754	0.029	-0.038	0.096	0.396
Model 1	10	0.639	0.528	0.750				
Model 2	10	0.633	0.519	0.747	-0.006	-0.077	0.065	0.871

Supplement Table S4B

Model 1: MELD ; Model 2: Peak VO2

model	*time*	*AUC*	*lower*	*upper*	*Delta_AUC*	*Lower_CI*	*Upper_CI*	*p_value*
Model 1	2.5	0.741	0.650	0.832				
Model 2	2.5	0.650	0.551	0.750	-0.091	-0.182	0.001	0.052
Model 1	5	0.651	0.563	0.738				
Model 2	5	0.616	0.526	0.707	-0.034	-0.114	0.046	0.400
Model 1	7.5	0.660	0.575	0.746				
Model 2	7.5	0.670	0.584	0.755	0.009	-0.067	0.086	0.810
Model 1	10	0.636	0.524	0.748				
Model 2	10	0.629	0.515	0.743	-0.007	-0.091	0.078	0.877
